# Efficacy and Tolerance of Post-operative Hypo-Fractionated Stereotactic Radiotherapy in a Large Series of Patients With Brain Metastases

**DOI:** 10.3389/fonc.2019.00184

**Published:** 2019-03-28

**Authors:** Geoffrey Martinage, Julien Geffrelot, Dinu Stefan, Emilie Bogart, Erwan Rault, Nicolas Reyns, Evelyne Emery, Samira Makhloufi-Martinage, Raphaelle Mouttet-Audouard, Laurent Basson, Xavier Mirabel, Eric Lartigau, David Pasquier

**Affiliations:** ^1^Academic Department of Radiation Oncology, Centre Oscar Lambret, University Lille II, Lille, France; ^2^Department of Radiation Oncology, Centre François Baclesse, Caen, France; ^3^Department of Biostatistics, Centre Oscar Lambret, Lille, France; ^4^Department of Medical Physics, Centre Oscar Lambret, Lille, France; ^5^Department of Neurosurgery, CHRU Lille, Lille, France; ^6^Neurosurgical Department, Universitary Hospital Caen, Caen, France; ^7^Department of Clinical Research and Innovation, Centre Oscar Lambret, Lille, France; ^8^CRIStAL UMR CNRS 9189, Lille University, Lille, France

**Keywords:** radiotherapy, hypofractionated stereotactic radiation therapy, brain metastasis, surgery, Cyberknife

## Abstract

**Purpose:** The aim of this study was to assess, in a large series, the efficacy and tolerance of post-operative adjuvant hypofractionated stereotactic radiation therapy (HFSRT) for brain metastases (BMs).

**Materials and Methods:** Between July 2012 and January 2017, 160 patients from 2 centers were operated for BM and treated by HFSRT. Patients had between 1 and 3 BMs, no brainstem lesions or carcinomatous meningitis. The primary endpoint was local control. Secondary endpoints were distant brain control, overall survival (OS) and tolerance to HFSRT.

**Results:** 73 patients (46%) presented with non-small cell lung cancer (NSCLC), 23 (14%) had melanoma and 21 (13%) breast cancer. Median age was 58 years (range, 22–83 years). BMs were synchronous in 50% of the cases. The most frequent prescription regimens were 24 Gy in 3 fractions (*n* = 52, 33%) and 30 Gy in 5 fractions (*n* = 37, 23%). Local control rates at 1 and 2 years were 88% [95%CI, 81–93%] and 81% [95%CI, 70–88%], respectively. Distant control rate at 1 year was 48% [95%CI, 81–93%]. In multivariate analysis, primary NSCLC was associated with a significant reduction in the risk of death compared to other primary sites (HR = 0.57, *p* = 0.007), the number of extra-cerebral metastatic sites (HR = 1.26, *p* = 0.003) and planning target volumes (HR = 1.15, *p* = 0.012) were associated with a lower OS. There was no prognostic factor of time to local progression. Median OS was 15.2 months [95%CI, 12.0–17.9 months] and the OS rate at 1 year was 58% [95% CI, 50–65%]. Salvage radiotherapy was administered to 72 patients (45%), of which 49 received new HFSRT. Ten (7%) patients presented late grade 2 and 4 (3%) patients late grade 3 toxicities. Thirteen (8.9%) patients developed radiation necrosis.

**Conclusions:** This large multicenter retrospective study shows that HFSRT allows for good local control of metastasectomy tumor beds and that this technique is well-tolerated by patients.

## Introduction

Brain metastases (BMs) are the most frequent brain tumors, and, throughout disease course, 20–40% of cancer patients will develop a BM (1). In subjects in good general health and presenting with a single BM, surgical resection has been shown to improve survival (2, 3). After surgery, adjuvant whole-brain radiotherapy (WBRT) allows to significantly reduce local and brain recurrence rates, as well as the risk of death from neurological cause (4, 5). Nevertheless, WBRT has not been shown to be beneficial in terms of overall survival (4–6) and the length of time in which patients remain functionally independent (4, 5). In addition it contributes, in the short term, to a poorer quality of life in patients (6) and causes acute toxicities including asthenia, alopecia, nausea, and a decline in learning and memory functions (7). Stereotactic radiosurgery (SRS) allows for good local control of the disease while avoiding the neurocognitive decline triggered by WBRT (8). Consequently, after resection of a BM, SRS and Hypofractionated stereotactic radiation therapy (HFSRT) are increasingly being used and could be considered as an alternative treatment standard to WBRT allowing to limit toxicity (7, 8).

To date, there is no consensus on the optimal dose, fractionation, or prescription regimens of HFSRT on the surgical cavity. Several prescription patterns are described in the literature, including schemas of 3 fractions with doses ranging from 7.7 to 11 Gy, (9–12) or schemas of 5 fractions (13, 14). Such heterogeneity in prescription doses prevents any direct comparison between studies. The largest phase III randomized study, comparing SRS to WBRT published by Brown et al. showed a longer cognitive-deterioration-free survival in patients assigned to SRS (median 37 months) than in patients assigned to WBRT (median 30 months) (*p* < 0.0001) (8). Overall survival (OS) was identical in the 2 arms, but local and distant brain control were lower in the SRS arm.

The aim of this study was to evaluate the efficacy and safety of post-operative HFSRT in resection cavity of secondary brain lesions in a large cohort of patients.

## Materials and Methods

### Population

Between July 2012 and January 2017, patients treated with post-operative HFSRT to the resection cavity in two French centers were included. Data were retrospectively collected. Inclusion criteria were: adult patients, with 1 to 3 BMs, no previous radiotherapy treatment to the brain, treated by surgery for BM of a solid tumor and with anatomical pathology data, no brainstem lesion or carcinomatous meningitis, eligible to be treated by HFSRT as decided in a multidisciplinary meeting, with a life expectancy of more than 3 months, and not opposed to the use of their medical data for research and educational purposes.

### HFSRT Technique

Patients were immobilized using a thermoplastic mask system. Computed tomography (CT) scan and gadolinium contrast-enhanced magnetic resonance imaging (MRI) were used for treatment planning. Imaging was performed using millimetric slices and rigid registration. Target volumes and organs at risk were contoured on MRI and concordance with CT was controlled. Contouring software's used were Oncentra (version 4.3.0) and Multiplan (Accuray, version 3.2.0).

Target volumes were contoured using the surgical and anatomical pathology assessment of resection specimens. The clinical target volume (CTV) included the surgical cavity, contrast enhancement of tumor border and a 1–2 mm margin which delineates it on the CT scan and planning MRI. In the case of metastasis in contact with dura, the CTV included a larger margin (5–10 mm) beyond the area where there was contact before surgery. The gross tumor volume (GTV) was defined if macroscopic disease could be identified by nodular contrast enhancement by T1-Gadolinum MRI imaging. The planning target volume (PTV) was defined as CTV + 1 mm.

HFSRT treatment was delivered using a CyberKnife®-type robotic accelerator (Accuray, Inc., Sunnyvale, CA), using 6 MeV photon beams, in Centre Oscar Lambret in Lille and Centre François Baclesse in Caen. Dose was prescribed at the 80% isodose and patients were treated every 2 days.

### Follow-Up

Follow-up of patients included collection of clinical data and brain perfusion MRI at 2 months and then every 3 months after the end of irradiation during the first year, and every 4 to 6 months thereafter. Local recurrence was defined as the appearance or growth of nodules in the surgical cavity visible on a T1-gadolinium MRI sequence. OS was defined as the time from HFSRT until death from any cause. Time-to distant brain control (DBC) was defined as to the time from HFSRT until progression in the brain outside of the surgical cavity. Radiation necrosis was diagnosed based on clinical, morphological, and metabolic criteria, and was validated by experts. MR spectroscopic imaging and 18F-DOPA PET (Positron emission tomography) were used to support the diagnosis if needed.

### Statistical Analysis

Patient and disease characteristics were analyzed using descriptive statistics. Quantitative variables were expressed as median and range. Survival curves were estimated using the Kaplan Meier method. For time to progression, patients were censored at the date of last news or date of death from any cause. Time interval for overall survival was calculated from the date of HFSRT to the date of death from any cause. Patients alive were censored at the date of last news. Patients were censored at day 1 in case of missing information on the event.

After having checked the proportional hazard assumption (Schoenfeld residuals), prognostic factors of survival were identified using a univariate Cox regression model. *Hazard Ratios* (*HR*) and the 95% CI as well as the calculated probability (*p*-value) were presented for each model. In cases of non-proportional hazards, the “restricted mean survival time difference” (RMSTD) was used (15). Significant variables at *p* = 0.10 in the univariate model were included in the multivariate Cox stepwise backward model analysis. The following factors were analyzed: sex, age, primary disease, primary histology, RPA (recursive partitioning analysis) score, DS-GPA (Diagnostic-Specific Graded Prognostic Assessment) score, controlled primary tumor, location of BM, extracranial metastasis status, number of BM, time between primary tumor and BM diagnosis, partial resection and gross total resection, interval time between surgery and HFSRT, dose of HFSRT, salvage WBRT, SRS or HFSRT, pre- and post-operative volumes, conformity index, and homogeneity index.

The association of the radiation necrosis with the different factors was analyzed with the Fisher exact test for qualitative variables and with the Wilcoxon Mann-Whitney test for quantitative variables.

Statistical analyses were performed using Stata 13.1 (StataCorp. 2013 Stata Statistical Software: Release 11. College Station, TX: StataCorp LP) and significance level was set at a *p*-value of 0.05 for all analyses.

## Results

### Population

This was a retrospective study involving 160 patients and 167 surgical cavities. Patients were 76 women (47.5%) and 84 men. The median age at diagnosis of BM was 58 years (range, 22–83 years) (Table 1). Seventy-three patients (46%) presented with primary lung cancer, 23 patients (15%) with melanoma and 21 patients (13%) with breast cancer. The median time interval between the primary tumor diagnosis and BM surgery was 8.4 months (range, 0–148.6 months).

**Table 1:** Patient characteristics.

**Patient characteristics**	***n***	**%**
**Patients**	160 (167 cavities)	
**Sex**	160/160	
Female	76	47.5%
Male	84	52.5%
**Age (y)**		
Median (range)	58	(22; 83)
**Primary disease**	157/160	
**NSCLC**	**73**	**46%**
**Cutaneous**	**23**	**15%**
**Breast cancer**	**21**	**13%**
Gastrointestinal	16	10%
Gynaecologic	9	6%
Renal cell carcinoma	6	4%
Other	9	6%
**Histology**	160/160	
**Adenocarcinoma**	**102**	**64%**
**Melanoma**	**24**	**15%**
**Squamous cell carcinoma**	**12**	**8%**
Other	22	14%
**Metachronous BM**	**78/157**	**50%**
**Synchronous BM:**		
Controlled systemic disease	63	40%
Uncontrolled systemic disease	16	10%
**Number of other extra BM sites**	157/160	
0	74	47%
1	52	33%
2	26	17%
≥3	5	4%
**RPA score**	156/160	
**1**	**75**	**48%**
**2**	**78**	**50%**
3	3	2%
**DS-GPA score**	134/160	
Median (range)	**3**	(1; 4)
**PS scale**	156/160	
**0**	**56**	**36%**
**1**	**86**	**55%**
2	13	8%
3	1	1%

*BM, Brain Metastases; DS-GPA, diagnosis-specific GPA; GPA, Graded prognostic assessment; NSCLC, Non-small cell lung cancer; RPA, Recursive partitioning analysis; PS Scale: Performance Status scale*.

### Description of BM and Treatment by HFSRT

At the time of diagnosis, 115 patients (72%) had a single BM; 77 patients (50%) had symptoms of intracranial hypertension and 126 patients (81%) had neurological symptoms (Table 2). Seventy-eight patients (50%) presented with synchronous BM, 63 patients (40%) with a metachronous BM and a controlled primary tumor, and 16 patients (10%) with a metachronous and a non-controlled primary tumor. Pre-operative MRI revealed a median tumor size of 32 mm (range, 7–78 mm) and 75% of the cases (*n* = 124) were supratentorial. Planning MRI was performed in 151 patients (94%). The median surgery cavity size was 27 mm (range, 5–66 mm) and in 46 patients (30%) a nodular contrast enhancement by planning MRI led to the diagnosis of an early relapse in the surgical cavity. Most frequent prescription regimens were 24 Gy in 3 fractions (*n* = 52, 33%) and 30 Gy in 5 daily fractions (*n* = 37, 23%). Median CTV and PTV volumes were 10.6 mL (range, 0.9–98.8 mL) and 15.2 mL (range, 2.2–129.8 mL), respectively.

**Table 2:** Brain Metastases and HFSRT treatment characteristics.

**Brain metastases and treatment characteristics**	***n***	
**Resection cavities treated**	167	
**Preoperative size**	136/167	
Median (mm)–(range)	**32**	(7; 78)
**Resected cavity size**	104/167	
Median (mm)–(range)	**27**	(5; 66)
**Location**	167/167	
Supratentorial	124	74%
Infratentorial	43	26%
**Synchronous BMs at time of HFSRT**	160/160	
None	115	72%
1	29	18%
2	16	11%
**Local relapse on planning MRI**		
No	107	70%
** Yes (nodule)**	**46**	**30%**
**Time between diagnostics and surgery**	161/167	
Median (months)–(range)	**0.4**	(0; 138)
**Time between surgery and CK treatment**		
Median (days)–(range)	59.5	(21;181)
**Gross total resection**	*n* = 117/127	**92%**
**Associated treatment during CK**	*n* = 68/139	**43%**
None	71	
Chemotherapy	30	
Targeted therapy	8	
Immunotherapy	8	
Anti-angiogenic	1	
*Unknown*	*21*	
**Delivered dose regimen**		
**24 Gy in 3 fractions**	**52**	**33%**
**30 Gy in 5 fractions**	**37**	**23%**
**27–30 Gy in 3 fractions**	**34**	**22%**
30 Gy in 6 fractions	15	9%
Other	22	14%
**Clinical target volume (CTV)**		
Median (cm^3^)–(range)	**10.6**	(0.9; 98.8)
**Planning target volume (PTV)**		
Median (cm^3^)–(range)	**15.2**	(2.2;129.8)
**D2 CTV**		
Median (Gy)–(range)	**33.6**	(25–50)
Mean (Gy) (±standard deviation)	**34 ± 5.3**	
**D50 CTV**		
Median (Gy)–(range)	**31.6**	(22–46)
Mean (Gy) (±standard deviation)	**32 ± 4.9**	
**D98 CTV**		
Median (Gy)–(range)	**30.2**	(20.7–43.1)
Mean (Gy) (±standard deviation)	**29.4 ± 4.6**	
**Brain V**_**12-Gy**_		
Median (cm^3^)–(range)	**53.2**	(4.0; 380)
**Brain V**_**21-Gy**_		
Median (cm^3^)–(range)	**22.9**	(0.01; 230)
**Brain D50**		
Median (Gy)–(range)	**1.3**	**(0.2; 6.2)**

*BM, Brain Metastases; CK, CyberKnife; DX, Dose received by x% of the volume of interest; MRI, Magnetic Resonance imaging; V_12−Gy_ and V_21−Gy_, Volume (cm^3^) of brain that received doses of 12 and 21 Gy*.

### Local Control

**Figure 1:**
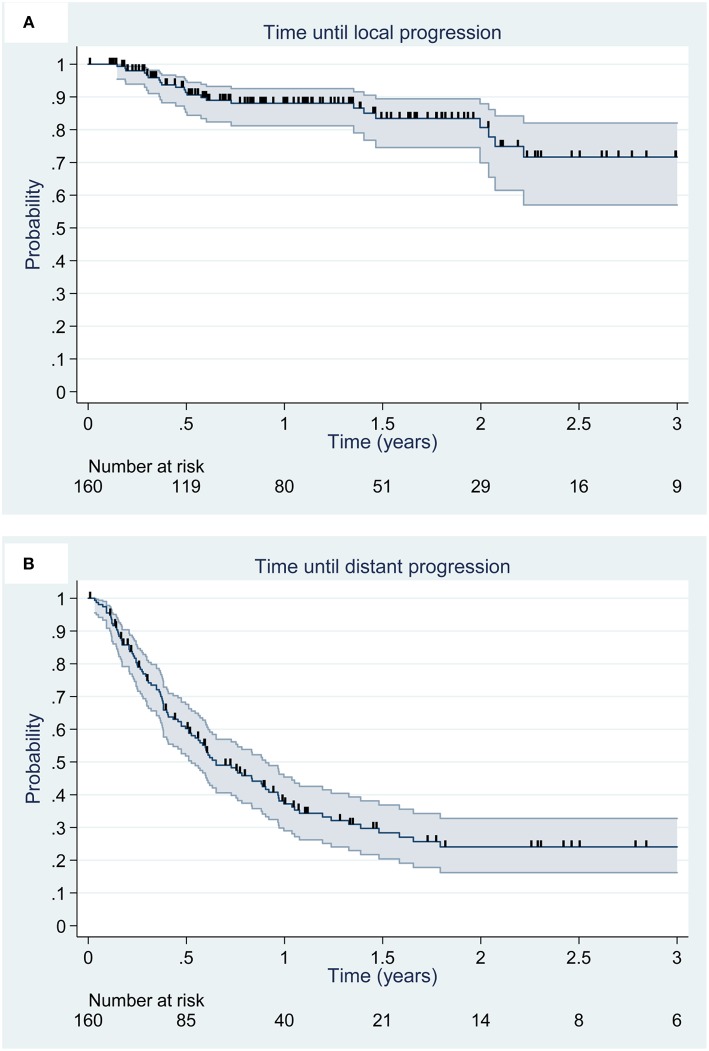
Kaplan Meier estimation (95% CI) of local **(A)** and distant **(B)** control. Deceased patients and patients alive at the date of last news were censored and are illustrated by vertical lines.

The median follow-up was 30.6 months. At the end of the follow-up, 23 local recurrence (14.4%) were observed. Local control rates at 6 months, 1 year and 2 years were 91% [95% CI, 85–95%], 88% [95% CI, 81–93%], and 81% [95% CI, 70–88%], respectively (Figure 1A). No factor appears to be prognostic of local control.

### Distant Brain Control

At the end of the follow-up, 86 patients (53.7%) presented with DBC. The median time to brain recurrence was 11.2 months (range, 8.4–18.0 months). DBC rates at 6 months, 1 and 2 years were 71% [95% CI, 63–78%], 48% [95% CI, 39–56%], and 34% [95% CI, 24–43%], respectively (Figure 1B). No factor appears to be prognostic of time to distant brain progression. The progression free leptomeningeal progression rate were 80% [95% CI, 73–86%] at 1 year and 72% [95% CI, 59–82%] at 3 years.

### Overall Survival

**Figure 2:**
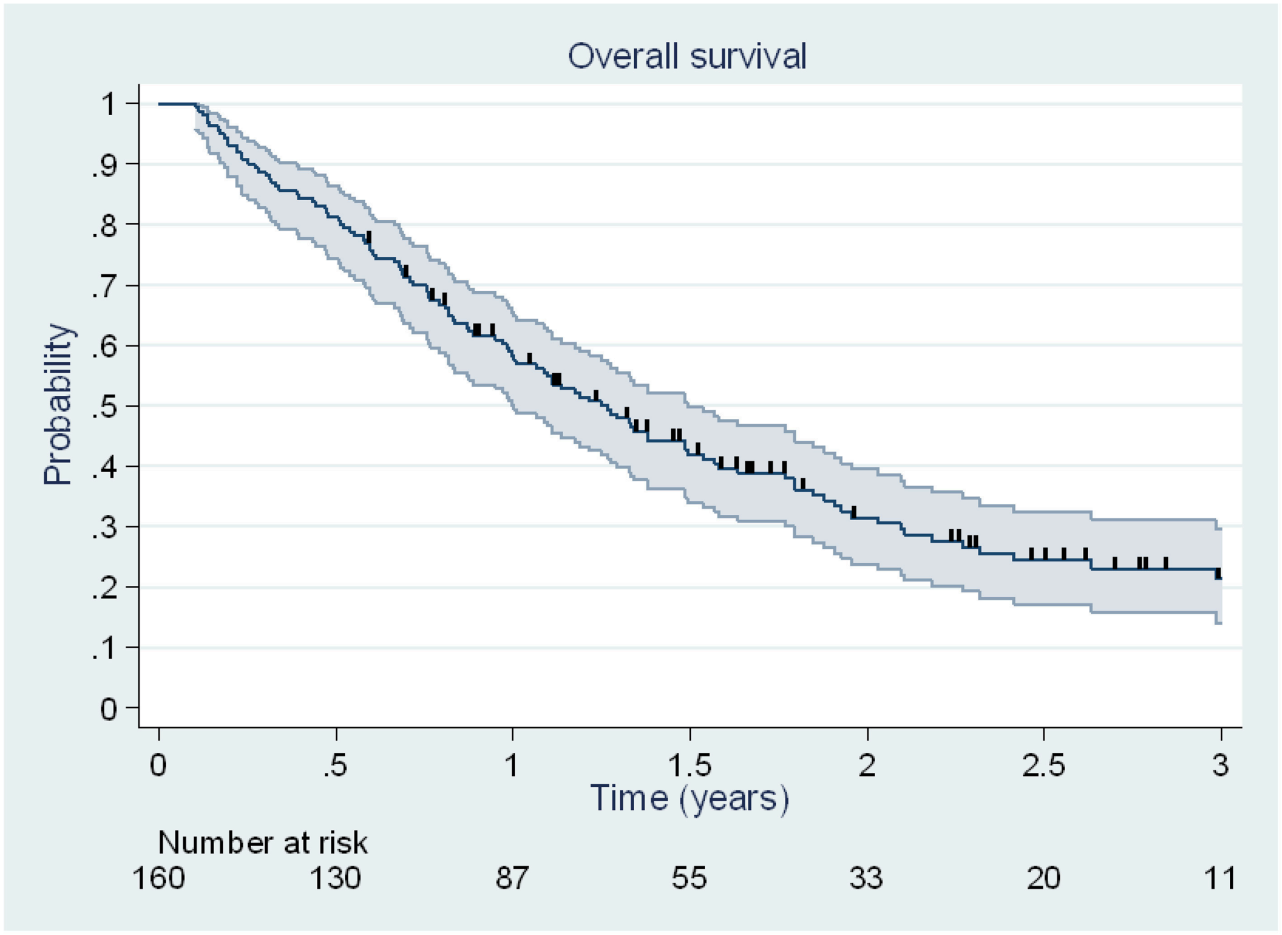
Kaplan Meier estimation (95% CI) of overall survival. Patients alive at the date of last news were censored and are illustrated by vertical lines.

At the end of the follow-up, 113 deaths (70.6%) were observed, including 33 deaths (42%) due to disease brain progression. Median OS was 15.2 months [95% CI, 12.0–17.9 months], and 6 months, 1 and 2 year OS rates were 81% [95% CI, 74–86%], 58% [95% CI, 50–65%] and 32% [95% CI, 24–39%] (Figure 2).

In the univariate analysis, different prognostic factors appears to be associated with overall survival. Lung primary tumor was associated with a significant reduction of the risk of death compared to other primary tumors (*HR* = 0.65, [95% CI, 0.44–0.94], *p* = 0.023). The number of extra-cerebral metastatic sites (*HR* = 1.19, [95% CI, 1.02–1.39], *p* = 0.027), the number of BM (*HR* = 1.17, [95% CI, 1.00–1.35], *p* = 0.046), the absence of systemic control of the disease (*HR* = 1.39, *p* = 0.035) and larger PTV (*HR* = 1.12, [95% CI, 1.01–1.26], *p* = 0.040) were associated with a significant increase of the risk of death. In multivariate analysis, lung cancer (*HR* = 0.57, [95% CI, 0.38–0.86], *p* = 0.007), the number of extra-cerebral metastatic sites (*HR* = 1.26, [95% CI, 1.08–1.48], *p* = 0.003) and the larger PTV (*HR* = 1.15, [95% CI, 1.03–1.28], *p* = 0.012) were prognostic of OS (Table 3). The number of BM did not achieve significance with a *HR* = 1.16 (*p* = 0.055).

**Table 3:** Prognostic factors of overall survival in multivariate analysis (Cox model).

**Predictive factors**	***HR***	**95% CI**	***p***
**Primary disease**			**0.007**
Other	1		
NSCLC	0.57	0.38–0.86	
**Number of other extra cerebral metastatic sites**	1.26	1.08–1.48	**0.003**
**Planning target volume**	1.15	1.03–1.28	**0.012**
**Number of BM**	1.16	0.99–1.35	**0.055**

*NSCLC, Non-Small Cell Lung Cancer; BM, brain metastases*.

### Salvage Treatments

Among the 23 patients presenting a local recurrence, 7 were treated by stereotactic re-irradiation, 6 by a WBRT, and 9 did not receive additional irradiation (Table 4). Overall, 72 patients (45%) underwent another brain irradiation: 38 (24%) received exclusively SRT at a median delay of 7.3 months (range, 1.3–58 months) and 34 (21%) received WBRT at a median delay of 7 months (range, 1.8–33 months). Sixty-six patients (41.8%) presented new neurological sign related to disease progression, at a median delay of 6.3 months (range, 0.9–32.2 months) after the initial radiotherapy treatment.

**Table 4:** Radiotherapy treatment for brain recurrence.

**Patient characteristics (*n* = 160)**	***n***	**%**
**Treatment of local recurrences**	**23**	
- WBRT	6	26%
- SRT	7	30%
- No re-irradiation	9	39%
- *Missing data*	*1*	4%
**Treatment of brain recurrences**	**72/160**	**45%**
- WBRT	23	14%
- SRT	38	24%
- WBRT + SRT	11	7%

*WBRT, Whole Brain Radiotherapy; SRT, Stereotactic Radiation Therapy*.

### Tolerance

According to the Common Terminology Criteria for Adverse Events (CTCAE) grading system, version 4.03, 5 patients (3.4%) presented an acute grade 2 toxicity and 1 patient (0.7%) presented an acute brain hemorrhage of grade 3. Ten patients (7.2%) developed late toxicity of grade 2 and 4 patients (2.7%) a late toxicity of grade 3 (two brain necroses, one seizure and one stroke). Radiation necrosis during follow-up occurred in 13 patients (8.9%). The stereotactic re-irradiation or WBRT was the only factor associated with an increased risk of developing a radiation necrosis (*p* < 0.001, Fisher exact test). Among patients that received HFSRT exclusively, the rate of radiation necrosis at the end of follow-up was 6.9% and among the 7 patients treated with stereotactic re-irradiation in the surgical cavity, 4 (57%) presented with a radiation necrosis.

## Discussion

This large series, the second largest after Keller et al.'s to our knowledge (12), shows that post-operative HFSRT to the surgical cavity of BM allows for a good local control with acceptable acute and late toxicity profiles.

### Local Control

Local control rates achieved in our series with HFSRT to the surgical cavity are comparable to those of the larger retrospective series. Keller et al. reported local control rates of 92.9% at 6 months, 88.2% at 1 year and 86.5% at 2 years in a series involving 181 patients and 189 surgical cavities (12) (Table 5). In this study, the prescribed dose was 3 × 11 Gy to the isocenter. Factors associated with a greater rate of local relapse in multivariate analysis were larger PTV (>24 mL), a greater GPA score and meningeal contact of the BM. Patel et al. and Mahajan et al. also demonstrated in their series that the tumor volume was predictive of local control (16, 19). The 1 year local control rate of 88% [95% CI, 81–93%] in our study is similar to the 85% revealed by the meta-analysis involving 629 patients treated by SRT to the surgical cavity (17).

**Table 5:** Post-operative HFSRT and SRS for brain metastases literature data.

**Trials**	**Study**	***n***	**Median OS**	**Local control**	**Distant Brain Control**
Brown et al. (8) (Post-operative SRS)	Phase III	98/194	12.2 months	6 months: 80% 1 year: 61%	6 months: 72% 1 year: 65%
Mahajan et al. (16) (Post-operative SRS)	Phase III	63/128	17 months	6 months: 85% 1 year: 72%	1 year: 42%
Gans et al. (17) (Post-operative SRS)	Review 14 studies	629	14 months	1 year: 85%	Median: 8.4 months
Ling DC et al. (18) (Post-operative SRS or HFSRT)	Retrospective	99	12.7 months	6 months: 84% 1 year: 72% 2 years: 55%	1 year: 36% Median: 7.9 months
Keller et al. (12) (Post-operative HFSRT)	Retrospective	181	17.3 months	6 months: 93% 1 year: 88% 2 years: 87%	6 months: 70% 1 year: 61%
Current study (Post-operative HFSRT)	Retrospective	160	15.6 months	6 months: 91% 1 year: 88% 2 years: 81%	6 months: 71% 1 year: 48% 2 years: 34%

*HFSRT, Hypofractionated stereotactic radiation therapy; OS, Overall survival; SRS, Stereotactic radiosurgery*.

The phase III study from Kocher et al. evaluated the combination of WBRT or SRS with surgery to treat 359 patients with 1 to 3 BMs (4). Of 160 patients treated with surgery, 79 patients were randomized to the observation arm and 81 to the adjuvant WBRT arm. The 2 years local control rate was 41% in the observation arm vs. 73% (*p* < 0.001) in the surgery and WBRT combination arm, close to the rates observed in our study. A randomized study, recently published by Brown et al., included 194 patients from 48 centers and compared radiosurgery and WBRT as adjuvant treatment (8). The surgical cavities had to be smaller than 5 cm. Patients treated by WBRT had 6 months and 1-year local control rates of 87.1 and 80.6%, respectively. In addition, this study showed weak local control rates in the radiosurgery arm (80.4% at 6 months and 60.5% at 1 year), well-below the local control rates obtained in the WBRT arm (*p* = 0.00068). The lower local control rate in this study could be explained by the weak dose delivered. Indeed, patients treated by SRS received 12 to 20 Gy in one fraction, while patients in the WBRT arm received 37.5 Gy in 15 fractions or 30 Gy in 10 fractions. Robbins et al. demonstrated in their study the use of radiosurgery to the surgical cavity as adjuvant therapy for resected BM that a marginal dose in SRS under 16 Gy was predictive of local control (20). Mahajan et al. reported in a phase III study the local control rates of 85% at 6 months and of 72% at 1 year after adjuvant SRS after surgery for patients with 1 to 3 BM (16).

### Early Local Recurrence

In our study, 46 (30%) patients presented a nodular contrast enhancement by planning MRI, even though resection was macroscopically complete in 92% of the cases. This diagnosis is difficult, and because RANO and RECIST 1.1 criteria are not adapted in the post-surgery setting, radiologists used heterogeneous methods for diagnosis (21, 22). In the study from Jarvis et al. before post-operative radiosurgery, 12% of the patients presented a local recurrence at 1 month. The early local recurrence rate was 37.5% at 1 month in patients with a subtotal resection (23). In these studies, the median delay between surgery and radiotherapy was 4–7 weeks (11, 13, 18), but could range from 18 days (14, 24) to 4.5 months (25).

### Distant Brain Control

In our series, DBC rates are comparable to those reported in the literature after SRT (12). The phase III studies from Mahajan et al. and de Kocher et al. reported similar distant brain control rates, 43% at 1 year and 58% at 2 years, respectively in the observation arms (4, 16).

In this study, leptomeningeal disease seems more frequent (5, 12, 26). In Keller et al. study 89.4% [95% CI, 85.0–93.8%] and 88.9% [82.2–91.9%] of patients did not developed leptomeningeal disease at 1 and 2 year, respectively (12). In Atalar's study, 161 brain metastasis resection cavities treated from 1998 to 2011 with post-operative SRS were retrospectively reviewed. One and 2 year rates of leptomeningeal disease were 13%, but until 34% at 1 year for breast cancer (26). In our study, the rate of leptomeningeal disease may have been overestimated as we also reported very moderate leptomeningeal disease on MRI.

### Overall Survival

Median OS of patients in our study was 15.2 months [95% CI, 12.0–17.9 months] and it was comparable to that observed in other studies. In the meta-analysis by Gans et al. median OS was of 14 months; 12.2 and 17 months in the randomized studies by Brown et al. and Mahajan et al., respectively, and of 12.7 and 17.3 months in the large retrospective series by Ling et al. and Keller et al., respectively (8, 12, 16–18).

In our study, patients presenting a primary NSCLC had a lower risk of death, with an *HR* of 0.57 [95% CI, 0.38–0.86], (*p* = 0.007), with respect to patients presenting other primary tumors. The risk of death increased also with the number of extra-cerebral metastatic sites at the time of diagnosis (*HR* = 1.26 [95% CI, 1.08–1.48], *p* = 0.003) and with larger PTV (*HR* = 1.15 [95% CI, 1.03–1.28], *p* = 0.012).

In the meta-analysis by Gans et al. a higher prevalence of single metastases in the cohort was the only factor associated with higher OS (*p* < 0.02) (17). The study by Keller et al. reported in multivariate analysis, that a RPA score of 3 (*p* = 0.02), piecemeal resection (*p* = 0.017) and an increased number of BMs (*p* < 0.001) were independent prognostic factors for a lower OS. Patients with multiple BMs had a risk of death 2.4 times greater than patients with a solitary BM (*p* < 0.001) (12). Kocher et al. randomized phase III study evaluating the interest of adjuvant WBRT found in a multivariate analysis that the only factors with a significant impact on survival with Performance Status (PS) ≤ 2 were the initial PS (0 vs. 2, *p* = 0.004) and the presence of macroscopic tumor outside the brain (absent vs. present *p* = 0.001) (4).

Based on 7 randomized studies of the RTOG and 2,350 patients treated for BMs, Barnholtz-Sloan et al. developed a nomogram to estimate OS in patients with BM (27). The model revealed that the primary site was predictive of OS, with breast cancer and lung adenocarcinoma being associated with improved survival. Contrary to previous studies, in our series, the survival of patients with NSCLC could be improved with treatments including immunotherapy, targeted therapy and third generation tyrosine kinase inhibitors (28).

### Safety and Radiation Necrosis

The tolerance was acceptable with 2.7 and 0.7% of patients presenting acute grade 2 and grade 3 toxicities, and 7.2 and 2.7% late grade 2 and 3 toxicities, respectively. These results are in line with the 10% toxicity rate after HFSRT revealed by the meta-analysis by Gans et al. (17). Risk of radiation necrosis has been shown to decrease with lower doses, greater number of fractions and smaller volume of the treated surgical cavity (29, 30). Eaton et al. demonstrated that for cavities bigger than 3 cm, treatment by radiosurgery was associated with a greater rate of radiation necrosis, with a *HR* = 3.81 [95% CI, 1.04–13.93], (*p* = 0.043) compared to treatment by HFSRT (29). The risk of radiation necrosis at 1 year was of 10.3% with HFSRT and of 19.2% after radiosurgery. In our study, 8.9% of patients presented a radiation necrosis during follow up and only re-irradiation was found to be predictive of radiation necrosis, possibly due to a lack of statistical power related to a low number of events. Median volumes of brain that received doses of 10 Gy (V_10Gy_), 12 Gy (V_12Gy_), and 21 Gy (V_21Gy_) were not found to influence radiation necrosis. In Minniti et al.'s study using a schema of 3 × 9 Gy, the rate of radiation necrosis was 7% at 1 year and 16% at 2 years (31). This study showed that V_24Gy_ was the only factor associated with radiation necrosis with a 16.8 mL threshold (*p* = 0.03).

In order to standardize practice, Soliman et al. recently published CTV contouring guidelines for SRS of completely resected cavity BM defined by 10 experts based on 10 clinical cases (32). Our delineation practices are in line with these guidelines. Improvements in local control can be achieved by adding a 2 mm margin around the resection cavity (33). The choice of 1 mm to define PTV is arguable. In our study, this PTV allowed to compensate for repositioning errors. In other studies a margin between 0 and 4 mm was more frequently used. However, in Gans et al.'s meta-analysis the use of a margin to define PTV did not allow to improve local control or OS (17).

### Limitations

This is a retrospective study and several irradiation schemas were used. Nevertheless, the prescription regimen at the 80% isodose was homogeneous. For 55% of patients a dose of 8, 9 or 10 Gy in 3 fractions was prescribed.

## Conclusion

This large retrospective multi-center study shows that, in our population of patients operated for BM, adjuvant treatment by HFSRT allows for good local control in the surgical cavity. This non-invasive technique was well-tolerated by patients. HFSRT is an efficient treatment option for patients with operated BM. The rate of distant recurrence and in particular leptomeningeal disease seems higher than the rate observed after WBRT. A close follow-up by MRI is necessary in patients with a high risk of intra-cerebral recurrence.

## Data Availability

All datasets generated for this study are included in the manuscript and/or the supplementary files.

## Ethics Statement

This study was carried out in accordance with the recommendations of local ethics committee and conducted in accordance with the Helsinki declaration with written informed consent from all subjects. All subjects gave written informed consent in accordance with the Declaration of Helsinki. The protocol was approved by the Centre O. Lambret ethics committee.

## Author Contributions

DP and GM contributed to the conception, design, and manuscript preparation. GM performed data acquisition. GM, JG, DS, EB, ER, NR, EE, SM-M, RM-A, LB, XM, EL, and DP performed data analysis and interpretation. EB carried out statistical analysis.

### Conflict of Interest Statement

The authors declare that the research was conducted in the absence of any commercial or financial relationships that could be construed as a potential conflict of interest.

## Abbreviations

HFSRT: Hypofractionated Stereotactic Radiation Therapy
BM: Brain Metastase
OS: Overall Survival
NSCLC: Non-Small Cell Lung Cancer
WBRT: Whole-Brain Radiotherapy
HFSRT: Hypofractionated Stereotactic Radiation Therapy
SRS: Stereotactic Radiosurgery
CT: Computed Tomography
MRI: Magnetic Resonance Imaging
CTV: Clinical Target Volume
GTV: Gross Tumor Volume
PTV: Planning Target Volume
DBC: Distant Brain Control
PET: Positron Emission Tomography
HR: Hazard Ratio
RMSTD: Restricted Mean Survival Time Difference
RPA: Recursive Partitioning Analysis
DS-GPA: Diagnostic-Specific Graded Prognostic Assessment
CTCAE: Common Terminology Criteria for Adverse Events.
